# Identification and Management of Atherosclerotic Cardiovascular Disease Risk in South Asian Populations in the U.S.

**DOI:** 10.1016/j.jacadv.2023.100258

**Published:** 2023-03-31

**Authors:** Anandita Agarwala, Priyanka Satish, Mahmoud Al Rifai, Anurag Mehta, Miguel Cainzos-Achirica, Nilay S. Shah, Alka M. Kanaya, Garima V. Sharma, Dave L. Dixon, Roger S. Blumenthal, Pradeep Natarajan, Khurram Nasir, Salim S. Virani, Jaideep Patel

**Affiliations:** a Center for Cardiovascular Disease Prevention, Baylor Scott and White Health Heart Hospital Baylor Plano, Plano, Texas, USA; b Division of Cardiovascular Prevention and Wellness, Houston Methodist DeBakey Heart and Vascular Center, Houston, Texas, USA; c Houston Methodist DeBakey Heart and Vascular Center, Houston, Texas, USA; d Johns Hopkins Ciccarone Center for the Prevention of Cardiovascular Disease, South Asian Cardiovascular Health Initiative (SACHI), Baltimore, Maryland, USA; e Pauley Heart Center, Virginia Commonwealth University, Richmond, Virginia, USA; f Institut Hospital del Mar d’Investigacions Mediques (IMIM), Barcelona, Spain; g Hospital del Mar, Parc Salut Mar, Barcelona, Spain; h Division of Cardiology, Department of Medicine, Northwestern University Feinberg School of Medicine, Chicago, Illinois, USA; i Department of Preventive Medicine, Northwestern University Feinberg School of Medicine, Chicago, Illinois, USA; j Division of General Internal Medicine, University of California San Francisco, San Francisco, California, USA; k Department of Pharmacotherapy & Outcomes Science, Virginia Commonwealth University, Richmond, Virginia, USA; l Cardiovascular Disease Initiative Broad Institute of MIT and Harvard, Cambridge, Massachusetts, USA; m Cardiovascular Research Center Massachusetts General Hospital, Harvard Medical School, Boston, Massachusetts, USA; n Center for Outcomes Research, Houston Methodist, Houston, Texas, USA; o Aga Khan University, Karachi, Pakistan; p Texas Heart Institute, Baylor College of Medicine, Houston, Texas, USA.

**Keywords:** diabetes, ethnic, lipid, prevention, risk, South Asian

## Abstract

South Asians (SAs, individuals with ancestry from Bangladesh, Bhutan, India, Maldives, Nepal, Pakistan, and Sri Lanka) are among the fastest growing ethnic subgroups in the United States. SAs typically experience a high prevalence of diabetes, abdominal obesity, and hypertension, among other cardiovascular disease risk factors, which are often under recognized and undermanaged. The excess coronary heart disease risk in this growing population must be critically assessed and managed with culturally appropriate preventive services. Accordingly, this scientific document prepared by a multidisciplinary group of clinicians and investigators in cardiology, internal medicine, pharmacy, and SA-centric researchers describes key characteristics of traditional and nontraditional cardiovascular disease risk factors, compares and contrasts available risk assessment tools, discusses the role of blood-based biomarkers and coronary artery calcium to enhance risk assessment and prevention strategies, and provides evidenced-based approaches and interventions that may reduce coronary heart disease disparities in this higher-risk population.

Cardiovascular disease (CVD) is the leading cause of noncommunicable disease burden globally and in the United States, contributing significantly to subsequent disability and rising health care costs.^1^ While successful primary and secondary CVD prevention campaigns have slowed the rate of CVD-related mortality, smaller decreases in heart disease death have been seen in some minority populations in the United States, including South Asians (SAs).^2-4^

South Asians (individuals who trace their ancestry from Bangladesh, Bhutan, India, the Maldives, Nepal, Pakistan, and Sri Lanka) are diverse with regard to region of origin, cultural identity, religious practices, cuisine, and language use. Accordingly, while the atherosclerotic cardiovascular disease (ASCVD) risk for SA adults is roughly double that of White adults,^5-7^ the risk of coronary heart disease (CHD) differs across SA subpopulations.^5^ Several cross-sectional studies have also reported a higher prevalence of prediabetes, type 2 diabetes (T2D), abdominal obesity, and hypertension, with lower levels of physical activity among SA adults compared to other racial/ethnic groups.^8-12^ The clinical presentation of ASCVD among SAs generally occurs earlier in life (mean age 53 years), with a higher burden of atherosclerosis, even in the absence of symptoms or clinical findings.^9,13-15^ Despite recognition of elevated ASCVD risk^16^ recommendations for risk, assessment, and stratification (a fundamental concept for the prevention of ASCVD), and subsequent management are not sufficiently tailored to the high ASCVD risk experienced by SA adults, largely because population-specific risk calculators inadequately estimate risk in this population, particularly for those at borderline or intermediate risk (≥5%-<20% by the pooled cohort equations [PCE]).^5,17^

Herein, we aim to address the roles of traditional and nontraditional risk factors, and review emerging strategies for risk assessment and reclassification, such as blood-based biomarkers and coronary artery calcium (CAC), that may better inform primary prevention of premature ASCVD in SAs who reside in the United States.

## SOUTH ASIANS ARE A HETEROGENOUS POPULATION

In the United States, understanding the health and disease patterns in specific Asian subpopulations has been challenging largely because Asian subgroups have frequently been aggregated into a single “Asian” category. The earliest SA immigrants (namely, from India and Pakistan) were regarded as ‘other’ when reporting US census data until 1920, followed by ‘Hindu.’^18^ The Luce-Celler Act of 1946 granted naturalization rights and extended immigration quotas allowing 100 people each from India and Pakistan to immigrate to the United States per year; these quotas were lifted in 1965, encouraging an influx of skilled professionals from all SA countries.^19^ Family reunification in the mid-1980s allowed further diversification of the SA population. Accordingly, the SA diaspora has spread across the United States with heavily concentrated pockets in California, the District of Columbia, Illinois, New Jersey, New York, Texas, and Virginia.^20^ The category ‘Asian Indian’ was the only SA subgroup identification available for immigrants on the US census card until 2010, after which ‘other Asian’ was introduced as an additional write-in category to encompass people from the remaining SA countries.^21,22^

In addition to more detailed U.S. Census and vital statistics race and ethnicity reporting, recent (Supplemental Table 1) and emerging (Supplemental Table 2a and 2b) research have focused specifically on SA health data. For example, CHD mortality rates are higher among SAs living in their native countries compared to those living in Western countries.^23,24^ Similarly, immigrant SAs in Europe have higher CHD rates compared to local populations.^78^ U.S. Asian Indian men and women have persistently higher age-standardized mortality rates from ischemic heart disease and heart failure when compared to non-Hispanic White (NHW) individuals.^3,6,25^ Compared to other SA subpopulations, however, the highest risk of CHD is seen among those of Bangladeshi origin, followed by Pakistani, then Indian adults: HR 3.66 (95% CI: 2.38-5.61), HR 2.45 (95% CI: 2.06-2.91), HR 1.83 (95% CI: 1.64-2.04), respectively.^5,26^ Other studies have demonstrated that Bangladeshi adults have the highest burden of diabetes, hyperlipidemia, and CHD among SA subpopulations.^27-29^

## TRADITIONAL RISK FACTORS

Traditional risk factors explain a large proportion of ASCVD risk in SA adults.^5,8,9,30,31^ The timing for screening among SAs compared to other ethnicities is less clear. ASCVD risk assessment guidelines in New Zealand suggest traditional risk factor (TRF) assessment in SA at age 30 years for men and 40 years for women, considering higher rates of CHD in immigrant SA living there.^30^ The US Preventive Service Task Force recommends lipid screening for men aged 20 to 35 and women 20 to 45, if they are at an increased risk of CHD (defined as: diabetes, history of previous CHD or atherosclerosis, family history of CVD, tobacco use, hypertension, and obesity (body mass index [BMI] ≥30 kg/m^2^)); however, they do not specify ethnicity/race.^32^

### TYPE 2 DIABETES

Compared to other racial/ethnic groups, SAs living in America have a high prevalence of T2D, albeit high variability exists by US state. The prevalence of T2D in SA according to the US National Health and Nutrition Examination Survey (2011-2016) was estimated at 22.4% compared to 12.1% in NHW, 20.4% in Black, and 22.1% in Hispanic adults, respectively.^33^ SA in the MASALA (Mediators of Atherosclerosis in South Asians Living in America) study (San Francisco and Chicago) showed a higher age-adjusted prevalence of diabetes compared to other adults in MESA (23% vs 6% in Whites, 18% in Blacks, 17% in Hispanic, and 13% in Chinese Americans).^11^ The age-adjusted prevalence of T2D using electronic health records (EHRs) for SA living in Northern California, New York City, and Atlanta were 29.1%, 10.7%, and 6.7%, respectively.^34,35^ The prevalence of T2D also varies among US and non-US community-dwelling SA subpopulations^36^: Bangladesh (10.4%-25%), Pakistani (11.6%-22.6%), Sri Lankan (7.8%-26.8%), Indian (7.1%-18.3%), Maldivian (7.6%), Bhutanese (4.9%), and Nepalese (3.0%-16.5%).^28,29,36-39^ Once diagnosed, residual poor glycemic control is more likely encountered compared to NHW, related to lack of culturally appropriate education, social stigmatization, beliefs about the need for diabetes medications, and uptake of traditional remedies, for example.^40-43^

National Health Interview Survey data suggest that Asian Indian adults were diagnosed with T2D 5 years younger (46 years old; 95% CI: 43.9-48.5 years), *P* < 0.001) than NHW (51 years old; 95% CI: 50.4-51.9 years), *P* < 0.001).^44^ Regional data using the California Health Interview Survey revealed that SAs were diagnosed with T2D 10.2 years earlier compared to NHW (mean age 44.9 vs 55.4, respectively).^45^

The high prevalence of diabetes is complex and multifactorial and is attributed to a combination of factors including excess visceral and intramyocellular adiposity and low lean muscle mass, obesity, metabolic syndrome, diet (traditionally vegetarian-but not all, high in fried carbohydrates, trans fat, and saturated fat), low rates of physical activity, high prevalence of low birth weight, pathophysiologic pathways including impaired insulin secretion and insulin resistance, and lifestyle and culture.^46-50^ Other metabolic abnormalities include higher plasma insulin levels, insulin-like growth factor-binding protein, and plasma leptin, and lower levels of adiponectin and resistin.^10,51,52^

South Asians are at high risk of T2D at a lower BMI compared to other ethnicities, even when accounting for other traditional risk factors.^40^ This is referred to as the ‘thin-fat’ or ‘South Asian’ phenotype of elevated fasting glucose, low high-density lipoprotein cholesterol (HDL-C), high triglycerides, and lower lean mass at normal ranges of BMI.^36,53^

Early and repeated screening for glucose intolerance and T2D may help identify at risk individuals. Indeed, there was a high incidence rate of glycemic progression in the MASALA study, where 32% (95% CI: 27.6-35.9) of participants progressed either from normal glucose tolerance to prediabetes or T2D, or from prediabetes to T2D over 5 years of follow-up.^36^ Recommendations from the American Diabetes Association (ADA), World Health Organization (WHO), National Institute for Health and Care Excellence (United Kingdom), and South Asian Health Foundation suggest that a BMI of ≥23 kg/m^2^ should trigger screening for diabetes in SA.^54-58^

### HYPERTENSION

In the United States, the age-adjusted prevalence of hypertension is 20 to 43% among studies of community-dwelling SA.^35,59-61^ These findings are consistent with the reported prevalence of 27% in a 2014 meta-analysis of observational studies from South Asian Association for Regional Cooperation member countries.^62^ Factors such as length of U.S. residency and poor dietary habits influence the development of hypertension.^49,63^

Hypertension is a well-established independent risk factor for myocardial infarction (MI), stroke, and chronic kidney disease.^8,64-66^ Compared to other racial groups/ethnicities, SAs have a higher rate of hypertension awareness, no difference in the rate of hypertension treatment, and lower rates of antihypertensive therapy adherence.^67,68^ Although outcomes data on ideal blood pressure (BP) goals, optimal medication regimen, and medication adherence are lacking, the guideline-recommended approach to aggressively treat BP is likely applicable. The most recent American College of Cardiology/American Heart Association (ACC/AHA) guidelines for the management of hypertension established new BP categories with lower treatment thresholds and BP goals.^69^ Indeed, 17% SA adults were recommended antihypertensive pharmacotherapy by the 2017 ACC/AHA hypertension guideline compared to 8% by Joint National Committee-7.^70^ The Blood Pressure Association UK Charity/South Asian Health Foundation has provided recommendations for managing hypertension in SA aimed at defining BP cutoff values, understanding the interplay of hypertension with other TRF, suggesting dietary and lifestyle enhancement, and explaining the value of pharmaceutical therapy if indicated.^71^ A summary of these recommendations is provided in Table 1.

### DYSLIPIDEMIA

Atherogenic dyslipidemia among SAs is characterized by higher levels of triglycerides and total cholesterol, lower/similar low-density lipoprotein-cholesterol (LDL-C), and lower levels of HDL-C compared to NHW.^95-97^ Compared to other racial/ethnic groups, SAs also may have higher levels of apolipoprotein (Apo)-B100 and non-HDL-C, lipoprotein(a) [Lp(a)], and low levels of Apo-A1, although more data are required.^9,98-100^ For example, in the INTERHEART study, Asian Indians had the lowest HDL-C, the highest ratios for total cholesterol/HDL-C and Apo-B/Apo-A1, respectively, across all LDL-C categories compared to other ancestral groups. Importantly, all of these parameters predicted future coronary artery disease, respectively.^23^

The prevalence of HDL-C <40 mg/dL in males and <50 mg/dL in females has been reported as high as 52% and 54% of Asian Indian men and women using EHR data in Northern California, respectively.^101^ The cardioprotective effect of HDL-C may be blunted in SAs compared to other East Asian subgroups: the OR for 1 SD increase in HDL-C among SAs was 0.87 (95% CI: 0.72-1.06), vs 0.77 (95% CI: 0.70-0.85) in other East Asians at the time of the first MI.^31^ This may be explained by a higher concentration of smaller HDL-C particles that contribute proinflammatory and prooxidant effects and are unable to participate in effective reverse cholesterol transport.^102,103^ Higher Apo-A1 (principal structural and functional protein component of HDL-C) levels in SAs are associated with a lower risk of MI.^31^

Lp(a) is genetically determined and highly atherogenic. Lp(a) is estimated to be elevated (>50 mg/dL or >125 nmol/L) in 25% of SA globally.^104,105^ The population attributable risk of MI was highest for SA when Lp(a) was >50 mg/dL.^106^ Compared to other racial/ethnic groups, the association of Lp(a) concentrations and MI in a case-control study was highest in SA (OR: 2.14, 95% CI: 1.59-2.89, *P* < 0.001).^106^ Prospective data suggest an association between elevated Lp(a) and ASCVD (HR: 1.31, 95% CI: 1.04-1.64, *P* = 0.023).^5^ Lp(a) levels were not associated with CAC prevalence (*P* = 0.98), common carotid atherosclerosis (*P* = 0.97), or aortic valve calcification (*P* = 0.64) in the MASALA study.^107,108^ SAs tend to have lower Lp(a) levels than Black adults, which may signal the need for ethnic specific Lp(a) thresholds to select truly higher risk individuals.^105,107^

South Asian men and women have similar or lower LDL-C levels compared to other racial/ethnic groups.^96,109^ Among SA subgroups, Pakistani adults had higher mean LDL-C values compared to North Indian (120 mg/dL vs 109 mg/dL, respectively; *P* = 0.02), although no difference was found between North and South Indian (*P* = 0.49) or between South Indian and Pakistani adults (*P* = 0.06).^110^ Importantly, at first time MI in the INTERHERT study, SAs has lower mean LDL-C compared to all other Southeast Asian subgroups (mean LDL-C 125.2 mg/dL vs 150.4 mg/dL, respectively). Compared to other Asian ethnicities, SAs have smaller, less dense LDL-C particles with a higher concentration of ApoB.^31^ This indicates a larger atherogenic particle load that may account for the elevated risk of ASCVD, even at lower LDL-C concentrations.^31,111^

In the absence of randomized controlled or prospective data, the National Lipid Association has provided expert opinion recommendations regarding the optimal primary prevention targets for lipid profiles in SAs, based on risk profile (high = PCE 10 years 20%-29%, very high = PCE 10 years 30%-39%, and extreme = PCE >40%).^88^ For example, LDL-C (mg/dL) goals for the 3 risk categories are <70, <50, and <30, respectively. The ideal triglycerides target is <150 mg/dL, and HDL-C (mg/dL) is suggested to be >40 in men and >50 in women.

### OVERWEIGHT AND OBESITY STATUS

Body composition and fat distribution are important determinants of CVD risk in SAs who tend to have a higher percentage of body, liver, and visceral fat compared to other ancestral groups.^10,112^ Hence, BMI calculations in SAs may be unreliable considering this body fat distribution. As such, the ADA and WHO recommended lowering BMI cut points to encourage public health action, with a focus on weight reduction and increased physical activity.^10,55,56^ Accordingly, the prevalence of obesity in SAs varies between studies. For example, in SA, the prevalence of obesity (defined as BMI ≥27.5 kg/m^2^) was 39.3% in men and 36.8% in women in a Northern California EHR cohort.^34^ National survey data indicated the overall prevalence of obesity may be higher at 77.6% (defined as BMI ≥23 kg/m^2^ in this study).^63^ National data also suggest that compared to other Asian subgroups, SAs have the one of the highest prevalence of overweight/obesity status.^113,114^

Compared with European Whites, SAs have a relatively greater amount of abdominal adipose tissue.^115^ Central adiposity is associated with insulin resistance and metabolic syndrome, contributes to hypertension, high cholesterol, lower HDL-C, and independently predicts acute ischemic heart disease in Asian Indians.^64^

Clinicians should be aware of cultural beliefs regarding body habitus considering self-perceived underestimates of weight status and the effect of weight on the risk for chronic diseases.^116,117^ Age at immigration and duration of residence in the United States are also correlated with a higher prevalence of overweight/obese status.^118,119^ SA infants have higher visceral and subcutaneous adipose tissue deposits compared to NHW, falsely capturing a healthy weight status even in the setting of higher metabolic risk.^120^ Primordial prevention efforts including a greater emphasis on maternal health during pregnancy may be necessary, considering SA children (ages 5-7) are more likely to be overweight/obese compared to children of other race/ethnicities.^121^

The ADA Diabetes Guidelines suggest annual BMI screening with a cut point value of ≥23 kg/m^2^ in SAs to define overweight status.^57^ In addition to encouraging a “healthy meal plan” and increased physical activity, obesity guidelines from the American College of Endocrinology also suggest annual screening (same BMI cut point as ADA) and define abdominal obesity in SA as a waist circumferences of ≥85 cm in men and ≥74 cm in women^57,78^; this is lower than SA waist circumference recommendations by the International Diabetes Federation Epidemiology Task Force Consensus Group (≥90 cm in men; ≥80 cm in women).^122^ The large-scale utility of other measurements such as adiposity (eg, bioelectric impedance, magnetic resonance imaging, and air/water displacement plethysmography) is limited considering the lack of outcome data.^78^

### DIET

As a result of urbanization, mechanization and increased availability of processed foods, SA countries and those who inhabit the diaspora have transitioned from diets rich in whole grains and complex carbohydrates to those that are higher in saturated fats and refined carbohydrates.^49,123-125^ In SA, a longer residence in the United States is directly associated with higher intake of saturated and trans fats, dietary cholesterol, and alcohol, for example (*P* < 0.05).^126^ This is consistent with the dietary habits of SAs after migration to European countries.^127^ Examples of deleterious cooking and dietary habits include: 1) high heat cooking and deep frying; 2) using reheated oil high in trans fats and advanced glycosylation end product; 3) using oils with high saturated fats (partially hydrogenated vegetable oil, palmolein oil); 4) lower quantity and quality of protein intake; and 5) high intake of sugar and refined carbohydrates.^128,129^

Alternatively, a diet higher in fruit, vegetables, nuts, and legumes is associated with a lower prevalence of hypertension and metabolic syndrome.^49^ A healthy plant-based diet was associated with a lower incidence of T2D, lower odds of fatty liver, and a better metabolic risk factor profile in MASALA.^85^ Additionally, a Mediterranean-type diet that incorporates traditional SA food is associated with a lower likelihood of obesity, fatty liver, and T2D.^85^ Higher attainment of cardiovascular health metrics (inclusive of a healthy diet) is associated with a lower prevalence of subclinical atherosclerosis as measured by CAC and carotid intima media thickness.^126^

Providing dietary recommendations requires a focus on providing culturally sensitive nutritional advice. For example, a relatively high proportion of Asian Indians follow a vegetarian diet, owing to religious beliefs or cultural reasons.^130,131^ Considerations must also be given to religious obligations such as Ramadan fasting, while encouraging balanced-meals (whole grains, fruits and vegetables, adequate hydration) during non-fasting hours.^132-134^ Dietary patterns vary among different SA communities. For example, a study of dishes from different parts of India showed a significant variation in fat and energy content.^135^ A South Asian food-specific carbohydrate counting tool has been developed to assist patients and providers understand the carbohydrate content of commonly consumed food products.^136^ The Canadian Heart and Stroke Foundation and National Lipid Association have also developed evidence-based dietary recommendations focused on portion control and informed choices, and are summarized in Table 1.^83,84^

### PHYSICAL ACTIVITY

When compared to other racial/ethnic groups living in North America, the prevalence of regular moderate physical activity is generally lowest in SA adults.^10,137^ A low-level of physical activity is independently associated with prediabetes and T2DM, obesity, and increased rate of death from CHD.^138-140^ Expectedly, regular moderate- or high-intensity exercise was protective for SAs in the setting of a first time acute MI in the INTERHEART study.^8^

Barriers to physical activity are variable and may be particular to a religion, gender, or generation. Low awareness of the benefits of physical activity, cultural gender norms (eg, modesty, healthy body weight perceptions), language barriers (poor English fluency), structural barriers (gender-segregation), perceived harm thresholds, career commitments, racial discrimination (institutional and personal), low levels of acculturation, communication gaps with health care professionals, and low self-efficacy have been cited as reasons for lower physical activity rates among SAs.^141-144^ Women are more likely to be sedentary compared to men, attributed to cultural expectations (restricted participation in some religious and ethnic groups; household responsibilities, child care, and supporting extended family members, for example).^145^

For all American adults, a minimum of 150 minutes of moderate-intensity exercise per week is recommended.^79^ Concerted efforts are needed to raise awareness of the benefits of physical activity and encourage culturally sensitive interventions via social networks and cultural/religious organizations.^80,146,147^ Interventions aimed at increasing neighborhood social cohesion, engaging community leaders, using community-based participation tailored to beliefs and norms, gender-specific measures and programs, encouraging youth sports participation, and culturally specific activities (Bollywood dancing and Bhangra, for example), may be effective measures to influence physical activity in immigrants and subsequent generations of SAs.^98,148-153^ Other specific cultural considerations for lifestyle modification are currently being explored; the SAHELI (South Asian Healthy Lifestyle Initiative) is a culturally targeted, community-based intervention designed to test the impact of lifestyle intervention to reduce the risk of CHD and T2D in SAs.^154,155^

### TOBACCO

The prevalence of tobacco use among American SAs is lower compared with other racial/ethnic groups, including other Asian ethnic groups.^156,157^ For example, the prevalence of current smoking was 12.4% among Filipinos, 5.9% among Chinese, and 18.5% among NHW compared with 5.1% among Asian Indians.^59,158^ In the MASALA study (with majority first-generation immigrants), 5% of men and 1% of women were current smokers. Commonly used questionnaires used to assess tobacco use typically do not capture cultural forms of tobacco, and therefore the prevalence of regular use of these products among SAs is not well understood and may be underestimated.^159^

In addition to combustible cigarette use, there are other forms of tobacco use specific to SA culture including smokeless (gutkha, naswar, paan, paan masala, zarda) and smoked products (bidi, hookah, shisha, chilam).^160,161^ Asking product-specific questions revealed high rates of alternative tobacco use in a New York City study among Bangladeshi and Gujurati adults.^162^ Tobacco use patterns may differ by gender as men were more likely to smoke while women were more likely to chew tobacco.^163^ SAs place emphasis on using culturally specific tobacco products during celebrations and social functions as a tribute to their heritage and a means of hospitality.^161,164^ There may also be inaccuracies in perceptions of the health effects and potential harms of tobacco products, which can further complicate their use.^164^

The prevalence of tobacco use may also differ among first generation compared with later generations. For example, among SA immigrants in the Northeast U.S., nearly half of female cigarette smokers were first-generation immigrants.^165^ Clinicians and public health officials should address the use and cardiovascular effects of these products using cultural-specific messaging. Available Center for Disease Control Asian-centric resources for smoking cessation are offered in non-South Asian languages.^166^ This is similar to other national antitobacco organizations.^167^ Pharmacotherapy and behavioral interventions to aid tobacco cessation should be offered to help curb use of these products, in line with national consensus recommendations.^75^

## NONTRADITIONAL RISK FACTORS

### FAMILY HISTORY

Family history of premature ASCVD (FamHx) (traditionally defined as age <55 for men and <65 for women) is a non-modifiable, established risk factor, that carries a temporal relationship for the development of future ASCVD (larger influence of shared, genetic component for premature events, and a more balanced contribution of environmental and acquired CVD risk factors for later onset events).^168^ Observational data suggest that the prevalence of FamHx in SAs ranges between 40% to 60%.^5,169-171^ The presence of a FamHx is associated with CHD, OR ranging from 1.45 (95% CI: 1.30-1.60) to 1.71 (95% CI: 1.21-2.42), depending on the study population and self-reported definition used.^5,170,172^ However, this is similar to other racial/ethnic groups.^169^ A one-time screening Lp(a) value in the presence of a FamHx may be useful to help further stratify ASCVD risk.^88,173^ A positive FamHx should promote a screening lipid panel for familial lipid disorders in adults older than age 20.^82^

### MARKERS OF INFLAMMATION

Inflammation is an important pathophysiological mechanism responsible for the initiation and progression of atherothrombosis.^174^ C-reactive protein is a marker of systemic inflammation that is typically elevated in Asian Indians compared with Whites and is associated with traditional risk factors and prevalent CVD.^175,176^ Other inflammatory biomarkers and adipocytokines of potential significance include homocysteine, tumor necrosis factor-α, leptin, and adiponectin. Plasma homocysteine levels are elevated among Asian Indians as compared with Whites and are associated with increased ASCVD risk.^177^ Dietary cobalamin deficiency is a plausible mechanism underlying homocysteinemia in this population although supplementation with vitamins B12, B6, and folic acid did not decrease the risk of CVD in the HOPE (Heart Outcomes Prevention Evaluation) 2 trial.^178,179^

The association of tumor necrosis factor-α, leptin, and adiponectin with cardiovascular risk among SAs remains to be studied. Alternatively, high-sensitivity C-reactive protein, tumor necrosis factor-α, leptin, and adiponectin were not associated with subclinical atherosclerosis as measured by CAC score in small studies highlighting a potential divergence in the pathophysiological role of inflammation and subclinical coronary atherosclerosis among SAs.^180^ Studies directly comparing risk estimation of markers of inflammation and CAC scoring in other racial/ethnic groups have reported the superiority of CAC in estimating future coronary disease events.^181-183^

### MATERNAL RISK FACTORS

Adverse pregnancy outcomes are related to an increased risk for ASCVD. Gestational diabetes mellitus (GDM) is a particularly important risk factor for ASCVD among SA American adults.^184^ Compared with NHW, non-Hispanic Black, Hispanic, and other Asian groups, nulliparous SA (Asian Indian) American women at first live birth experienced the highest rate of GDM in 2019 (129.1 per 1,000 live births), with a significant 4.4% per year increase in GDM rates between 2011 and 2019.^185^ Between 2014 and 2019, GDM rates were higher among Asian Indian women born outside the United States (122.7 per 1,000 live births) compared with those born in the United States (75.5 per 1,000 live births).^186^ SA American women in the MASALA study who reported a history of GDM had 3.2 times higher odds of having T2D, compared with women without GDM.^187^ Given the disproportionate burden of GDM experienced in SA American women, the American College of Obstetrics and Gynecology recommends screening for GDM early in pregnancy for SA (and other Asian American) women.^90^ GDM is known to have consequences for both the mother and offspring, although the available data are generally not from South Asian populations. GDM increases the risk of subsequent ASCVD in midlife^188^ and also increases the risk for premature ASCVD in the offspring.^189^ Confirming these findings and quantifying the magnitude of risk conferred among SA women remains to be evaluated.

Hypertensive disorders of pregnancy (HDP), which include pregnancy-induced hypertension and preeclampsia, are another important adverse pregnancy outcome that increases risk for ASCVD. In 2019, HDP rates among nulliparous Asian Indian women at first live birth were 54.1 per 1,000 live births, and HDP rates increased on average 9.0% per year between 2011 and 2019.^190^ HDP rates were higher among Asian Indian women born inside the United States (64.4 per 1,000 live births), compared with Asian Indian women born outside the United States (52.9 per 1,000 live births).^186^

While HDP is associated with a higher risk of subsequent premature mortality among other populations in the United States,^191^ the role of HDP and several other adverse pregnancy outcomes on the risk for ASCVD in SA American adults remains to be characterized. Differences in adverse pregnancy outcomes including GDM and HDP in subgroups of SA Americans (eg, Pakistani, Bangladeshi) have not been evaluated to date.

### BARRIERS TO ACCESSING HEALTH CARE

As the SA population in the United States grows, public health strategies must adapt to meet their needs.^192^ Identifying a racial group as “high risk” may serve to improve and/or provide health care resources to vulnerable populations, but also has the potential to thwart health-seeking behavior, engagement in care, and adherence to therapy.^193^ Furthermore, it does not account for disparities in care provision and process, socioeconomic position, neighborhood environment, sociocultural factors, and racial discrimination.^2,194^ Specific health challenges that impact cardiovascular health in SA and proposed opportunities to overcome these barriers are listed in Table 2.

ASCVD risk management considerations for South Asians are depicted in the Central Illustration and described in Table 1.

## RISK ASSESSMENT

Risk assessment is fundamental for ASCVD risk reduction counseling. However, risk prediction, discrimination, and calibration in SA adults remain challenging for the following reasons: 1) available guidelines recommended risk algorithms have not been derived from or prospectively validated in SA adults; 2) limited considerations have been given for native vs migrant populations; and 3) there is paucity of disaggregated data, which masks meaningful ASCVD health differences in SA subgroups. As such, available population-specific risk assessment tools unreliably estimate risk in SA at large and among subgroups^5,17,27,214-221^ (Table 3).

For example, the National Institute for Health and Care Excellence (NICE) guidelines recommended a crude adjustment factor to the Framingham Risk Score (FRS) for SAs (FRS multiplied by 1.4 for men; no recommendation for women).^241^ The FRS and UK Prospective Diabetes Study underestimates risk in SAs compared to a White European population.^240^ QRISK2 underestimates risk in SA women.^233^ The 3rd Joint British Societies’ CVD risk calculator accounts for SA ethnicity; however, underestimates risk considering the small proportion (2.3%) of SA adults included in the cohort.^232^

QRISK3 (5.25% SAs), ETHRISK (46% SAs), NORRISK-2 SADia (12% SAs) are not yet incorporated into any national guidelines.^238,239^ The 2019 WHO risk charts (estimates risk in Bangladesh, Bhutan, India, Nepal, and Pakistan) misclassify higher-risk SAs to low-risk categories.^242^ The INTERHEART Modifiable Risk-Score has been internally and externally validated for the prediction of future ASCVD risk in SAs; however, its application is limited considering case-control data used to develop the risk calculator.^236^

The 2013 AHA/ACC PCE recommend using “White” race for SA adults, resulting in risk underestimation.^17,222^ The 2016 European Prevention guidelines and 2018 American Blood Cholesterol guidelines introduced SA ethnicity as an ‘ASCVD risk-enhancer’ when considering the initiation of statin therapy.^82,243^ However, substantial variability exists in the CVD prevalence, incidence, risk, and health-seeking behavior among SA subgroups.^6,25,29,244-246^ Studies conducted among SAs in Europe highlight an overall increased CVD risk among SAs, but also demonstrate heterogeneity of risk by country of origin, generation of immigration, and acculturation.^98^ While European observations appear to mirror those observed in SAs residing within the United States, additional studies are imperative to filling in knowledge gaps that exist within this group.

## CORONARY ARTERY CALCIUM

Considering unreliable ASCVD-risk estimation methods and the absence of a validated SA-specific risk calculator, the use of CAC may be a useful test to improve risk stratification and guide primary preventive efforts.^17^ CAC testing is a cost-effective, highly reproducible, and specific marker of subclinical atherosclerosis.^247^

SA men and women with a 10-year predicted risk of >7.5% by the PCE were found to have a high CAC burden.^59^ Although formal validation is required, the PCE may adequately predict risk among SAs at low risk and high risk (<5% and >20%).^248^ However, the extent of ASCVD-risk overestimation using the PCE was greater among SA adults considered at low- and intermediate-risk compared to among NHWs.^17^ For example, intermediate-risk SA have a 73% higher odds of CAC = 0 (low short-term risk strata) compared to NHWs (95% CI: 1.00, 2.99). When considering SA ethnicity as a ‘risk-enhancing factor’ according to the 2018 AHA/ACC Blood Cholesterol Guidelines (ie, systemic statin pharmacotherapy considerations for borderline and intermediate risk SA adults), an absence of CAC (CAC = 0) was found in 54% and 30% of participants at borderline risk and intermediate risk, respectively.^82,248^

### ADVANCED CORONARY ARTERY CALCIUM MEASURES: BEYOND THE AGATSTON SCORE

Advanced CAC measures such as vessel involvement, density, and volume in SAs are summarized in Table 4.^169,170,248-253^ Overall, ASCVD event data are required to confirm the importance of these findings.^254-258^

### CORONARY ARTERY CALCIUM FOR PERSONALIZING PREVENTIVE THERAPIES

Coronary artery calcium (CAC) scoring may also guide safe allocation of other preventive pharmacotherapies. Considering the 2017 ACC/AHA Blood Pressure guideline, CAC scoring may help identify those SA adults who would best qualify for aggressive lifestyle optimization and antihypertensive pharmacotherapy.^69,70^ For example, the proportion of SA participants that would qualify for antihypertensive pharmacotherapy per the ACC/AHA, but not by JNC7 guidelines, was higher among those with CAC >100.^70^ In other racial/ethnic groups, CAC imaging has demonstrated the potential to inform the intensification of blood pressure management.^259-261^ Considering a high prevalence of prediabetes (not an indication for statin therapy) in SA adults, the presence of CAC may also influence statin initiation; alternatively if CAC = 0, statin therapy may be deferred/avoided in lieu of ongoing prudent lifestyle interventions.^88,248,262^

Considering the interplay of TRF and CAC and risk of early ASCVD events, an emphasis on the simultaneous appraisal of TRF and CAC in SA may be of clinical importance.^263^ In adults ≤45 years old of other racial/ethnic groups, the presence of CAC increases with the number of TRF, OR: 4.5 (95% CI: 2.7-7.3), in patients with >3 vs 0 TRF.^253^ Notably, SAs and NHW men have similar CAC burden (men age 58 ± 9 years vs 63 ± 10 years) but higher CAC burden compared to other racial/ethnic groups (mean age 62 ± 10).^109^ SA men have similar rates of CAC progression to NHW (interscan time 4.8 ± 0.8 years).^264^ These data are consistent with other studies showing similar prevalence and severity of CAC between Asian Indians and Whites.^265-267^

Among those with CAC = 0 at baseline, the timing of repeat CAC scanning has not yet been defined specifically for SAs; however, a 3- to 5-year interval has been suggested for those at borderline or intermediate risk by the PCE, and 3 years for those with diabetes.^268^ Notably, among SA adults with no CAC at baseline in the MASALA study, the age-adjusted CAC incidence was 8.8% (95% CI, 6.8%-10.8%) in men and 3.6% (95% CI, 2.5%-4.8%) in women on repeat CAC measurement after 4.8 ± 0.8 years.^264^ Despite favorable outcome data in the absence of CAC,^247^ a potential limitation of the CAC score lies in its inability to detect the entire spectrum of plaque morphology and burden.^269^ Coronary computed tomography angiography (CCTA) can evaluate coronary anatomy, stenosis, and characterize atherosclerotic plaques beyond the ability of CAC. Among multiethnic, asymptomatic populations that did not include SA, the prevalence of noncalcified plaque by CCTA ranges between 5.5% and 16% in patients with no CAC (% participants CAC = 0, 23%-59%)^269-272^ (NCT03920176). Although long-term outcome data are expected to confirm the clinical significance of these findings, a clinical practice statement from the American Society for Preventive Cardiology suggests judicious use of CCTA as an alternative to CAC in *asymptomatic* high-risk populations (eg, family history of premature ASCVD, familial hypercholesterolemia, diabetes, and those of SA descent with strong family history among others).^273^ Other major scientific societies have not embraced CCTA for ASCVD risk assessment in asymptomatic patients, highlighting the role of early and aggressive risk factor identification and management as a reasonable approach. This may be particularly applicable to those at the highest ASCVD risk, such as low-income, low-education, low rates of acculturation, and those with poor access to health care, for example.

In many of the aforementioned studies of CAC, the majority of SAs were Asian Indian (a lower-risk SA subgroup compared to other SA subpopulations such as Pakistani and Bangladesh, particularly those living in the United States), yielding caution to the widespread use of CAC for risk stratification until clinical outcome data are available.^5^ However, in other racial/ethnic groups, CAC is proven to improve ASCVD risk assessment, thereby serving as a guide for initiating or deferring preventive therapies.^247^ CAC scoring has shown promise with respect to further ASCVD risk refinement in SA adults as suggested by available consensus recommendations, particularly for Asian Indian adults.^88,180,262^

## CONCLUSIONS

ASCVD risk among South Asian adults in North America is heterogeneous and must be individualized. Awareness and management of traditional risk factors remain essential. Available risk stratification tools have their limitations, however the use of clinical tools, including blood biomarkers and CAC, may help transcend these limitations and personalize care. The difference in risk factor profiles and CVD phenotypes, and contribution of genetic susceptibility, environmental influences, and health-related behavior to this observed heterogeneity will be available as the MASALA, MASALA-2G, and OurHealth studies advance.^152,274,275^ Further characterization of national and cultural heterogeneity, the effect of immigrant duration, ASCVD risk in second- and third-generation SA subgroups, the effect of ethnically mixed families on cardiovascular risk profiles, and coronary vasculature characteristics (ie, vessel dimensions and subclinical atherosclerosis assessment), for example, represent remaining knowledge gaps for immigrant SAs living in European countries that are equally applicable to North American SAs.^79^ Education for patients and the healthcare team at large on navigating cultural, religious, social, geographic, and economic barriers is essential to delivering high-quality care.^72^ From a health policy perspective,^276^ it is imperative that the health needs of this group are addressed to ensure culturally appropriate medical and health services as a means of mitigating cardiovascular risk in this higher risk population.

## Supplementary Material

Supplemental Material

## Figures and Tables

**CENTRAL ILLUSTRATION:**
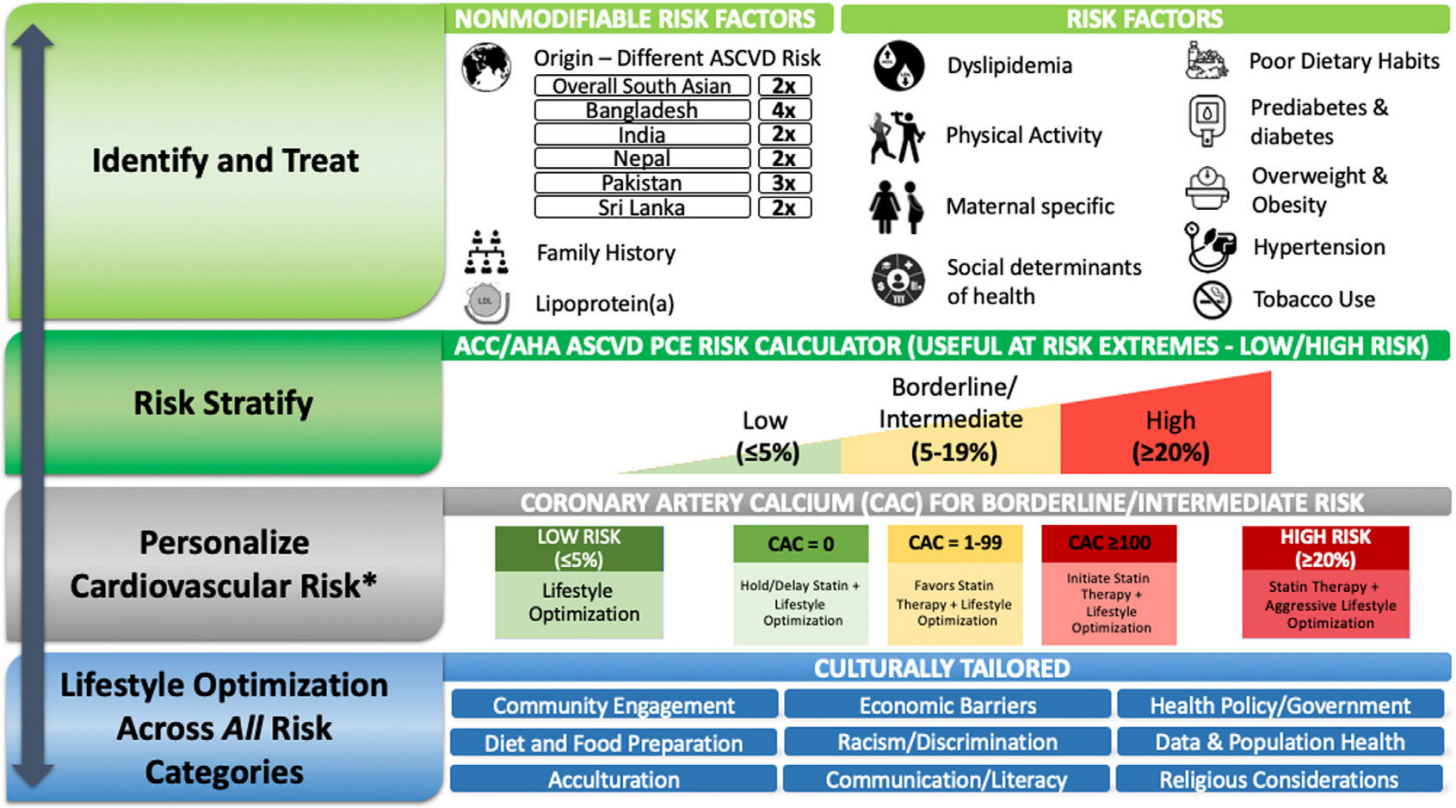
Primary Prevention Cardiovascular Risk Assessment and Management Considerations for the South Asian Populations in the United States *Data available mostly for Asian Indian adults.

**TABLE 1:** Primary Prevention Management Considerations for Individuals of South Asian Ancestry^72^

Step 1	Inquire about country of origin (ASCVD risk is highest among those from Bangladesh and Pakistan) and length of residency in America, if applicable.	
Step 2	Assess key risk factors.	
	Risk factor	Screening/Testing, if applicable.
	Prediabetes and type 2 diabetes	Screening suggested at 35 y of age according to available National guidelines^57^ Obtain fasting glucose level, at any body mass index^73,74^
	Hypertension	Screen for other ASCVD risk factors in adults with hypertension according to National guidelines^69,73^: smoking, diabetes, dyslipidemia, excessive weight, low fitness, unhealthy diet, psychosocial stress, and sleep apnea; testing may include fasting blood glucose, complete blood cell count, lipids, basic metabolic panel, thyroid-stimulating hormone, urinalysis, electrocardiogram with optional echocardiogram, uric acid, and urinary albumin-to-creatinine ratio.
	Dyslipidemia Common patterns: 1) ‘Atherogenic dyslipidemia,’ characterized by: ↓ HDL-C, ↑ triglycerides, and ↑ total cholesterol; 2) ‘HDL paradox’ defined as dysfunctional HDL-C, even at higher values.	In setting of increased risk of coronary heart disease, screen for lipid disorders in men 20-35 y old and women 20-45 y old according to available National guidelines^32^; consider testing for Lp(a), and/or apolipoprotein B-100 for further ASCVD risk stratification. Emphasize diet and lifestyle modifications.
	Tobacco use	Inquire about traditional (eg, cigarettes, cigars, electronic cigarettes, snuff) and cultural tobacco products: smokeless (eg, gutkha, naswar, paan, paan masala, zarda) and smoked (eg, bidi, hookah, shisha, chilam). Encourage cessation using pharmacotherapy and behavioral interventions in line with National guideline recommendations.^73,75,76^
	Overweight and obesity	Fasting blood glucose; screen for metabolic syndrome; target BMI ≤23 kg/m^2^. Management can include behavioral, pharmacologic, and surgical interventions if indicated.^77,78^
	Physical activity	Encourage a minimum of 150 min of moderate-intensity exercise per week,^79^ tailored to beliefs, norms, and modesty; consider gender-specific programs and culturally specific activities. Walking is a common form of exercise and should be encouraged (including with spouse, family, and friends, for example).^80^ A reasonable walking goal is 7,000-10,000 steps per day.^81^
	Family history of coronary artery disease (premature or any first-degree relative)	Screening lipid panel; selective lipid screening on the basis for family history in children and adolescents^82^; consider testing for Lp(a).
	Diet and nutrition	Provide culturally tailored educational material; emphasize moderation and encourage a diet rich in healthy plant-based foods (ie, whole grains, fresh fruits and vegetables, nuts, seeds, lentils, and legumes), liquid plant-based oils (ie, olive, canola, sunflower, and soybean), foods containing polyunsaturated fats (omega-3 and omega 6), viscous fibers (ie, oats, barley, flax seeds), and lean protein (ie, beans, legumes, fish, and chicken)73.^83-89^ Refer to nutritionist specializing in South Asian cuisine/diet, if available.
	Women’s reproductive health	Inquire about a history of gestational diabetes, hypertensive disorders of pregnancy (preeclampsia, eclampsia), or polycystic ovarian syndrome.^90-92^ Screen for diabetes if gestational diabetes was present. Obtain and manage blood pressure per available National guidelines.^71^
Step 3	Offer languages concordant with the patient’s ethnicity (eg, translator service), provide educational material written and translated into languages native to South Asian countries, provide and encourage education on cultural beliefs and practices for all team members of the healthcare team.	
Step 4	Risk assess: Calculate cardiovascular risk using the 2013 PCE; ‘South Asian’ ethnicity is a risk-enhancing factor for those at borderline and intermediate risk by the PCE.	
Step 5	Risk stratify: Consider use of CAC scoring to further stratify risk, particularly for those at borderline or intermediate risk (≥5%-<20% by the PCE), or those who are low risk by the PCE (<5%) with a family history of coronary heart disease.^82,88,93,a^	
Step 6	If available, refer to a South Asian cardiovascular and metabolic specialty program.^94^	

a Particularly for Asian Indian adults based on available data.

ASCVD = atherosclerotic cardiovascular disease; BMI = body mass index; CAC = coronary artery calcium; HbA1c = hemoglobin A1c; HDL-C = high-density lipoprotein cholesterol; PCE = pooled cohort equations.

**TABLE 2:** Barriers and Opportunities to Improve Cardiovascular Health in South Asian Adults

Barriers	Comments
Racial and cultural discrimination^195,196^	Racial/cultural discrimination experienced when seeking health care services may be related to poor self-rated health status and quality of life.^197,198^
Existing cultural attitudes regarding health care	Fatalistic beliefs (all events are predetermined and therefore inevitable), cultural, and social norms influence self-management and illness beliefs.^199,200^
Acculturation	In MASALA, 3 acculturation strategies were identified: separation (preference for South Asian culture over US culture), assimilation (preference for US culture over South Asian culture), and integration (similar level of preference for South Asian and US cultures). Length of stay in the US, English proficiency, and higher household income influenced assimilation or integration strategies.^201^ Those in the integration and assimilation strategies had better cardiometabolic risk factors than those in the separation class.^202^ Higher levels of acculturation influence health-seeking behaviors and higher self-reported health in a positive fashion.^203^
Socioeconomic status	Lack of health insurance and high out-of-pocket expense for appointments and prescriptions.^204^
Health literacy	Health literacy is closely associated with English proficiency and cultural health beliefs; limited literacy associated with a poor understanding of available health services, underutilization of available medical care, and lower levels of self-rated health status and diabetes care in SAs.^205,206^
Geography - distance from healthcare center, extended wait times, child care responsibilities, and lack of access to transportation	Transportation is especially problematic for elders who cannot drive or/and speak English and those who live in multigenerational homes.^207^
Language^207,208^	Effective healthcare use requires native language accessibility to explain symptoms, and understand diagnosis and treatment, for example. Children can help facilitate interpretation; however, this method is prone to incorrect or incomplete communication when children lack the appropriate medical terminology, lending itself to delay of care in the elderly.^209^ Lower rates of English proficiency are a predictor for higher traditional health practices.^210^
Health care practices do not align with modern Western or allopathic medicine	South Asian patients question the need and efficacy of modern medication. In parallel with medical therapy use, they may use traditional remedies, perceived to be more efficacious and nontoxic. Family and friends can serve as important in decisions to use alternative medicines.^43^
Opportunities	Comments
Culturally sensitive educational materials	Education material is written and translated into common languages native to South Asian countries. Educational material offers culturally specific information (eg, dietary recommendations that name foods common to a SA diet, or healthcare recommendations during religious obligations such as Ramadan fasting)^83,134^.
Language/Health literacy	Patient experience is improved in practice settings that offer a language concordant with the patient’s ethnicity.^208^
Engagement of cultural/religious organizations and social networks.	Partnering with places of worship or community events to promote healthy lifestyle education, healthy eating habits, increased physical activity, and combat the effect of discrimination and racism.^148,211,212^
Cultural competency	Systemic education on cultural behaviors and practices for all members of the healthcare team.^213^
Disaggregation of health data	Disaggregation will help refine our understanding of health care disparities among South Asian subpopulations, considering broad genetic, cultural, and socioeconomic characteristics.^3^

MASALA = Mediators of Atherosclerosis in South Asians Living in America; SA = South Asians.

**TABLE 3:** Select Risk Assessment Calculators and Guideline Considerations Specific to South Asian Adults

Risk Calculator	Population, Country	Number of South Asians in Derivation Cohort (%)	Guideline	Guideline Comments^a^
Pooled cohort equations (PCE)^222^	Pooled dataset of cohort studies, USA	0%	2018 ACC/AHA Blood Cholesterol Guidelines^82^ 2019 ACC/AHA Guideline on the Primary Prevention of Cardiovascular Disease^223^	South Asian designated as “White.” South Asian is considered a “risk-enhancing factor:” Class IIa recommendation (where benefit >> risk) for statin considerations in South Asian adults at estimated borderline and intermediate risk.
Systematic COronary Risk Evaluation (SCORE)^224^	Pooled dataset of cohort studies, Europe	0%	2019 ESC/EAS Guidelines for the Management of Dyslipidemia.^225^ 2021 ESC Guidelines on cardiovascular disease prevention.	Multiply risk by 1.3 for Indians and Bangladeshis, and 1.7 for Pakistanis. ‘Other Asian’: multiply risk by 1.1.^226^
Framingham Risk Score (FRS)^227^	Population cohort, USA	0%	2012 Australian Absolute CVD Risk Guidelines/National Vascular Disease Prevention Alliance^228^ 2021 Canadian Cardiovascular Society guidelines for the management of dyslipidemia^229^	Australia: for moderate risk (FRS 10-15% absolute risk), consider blood pressure and/or lipid lowering in addition to lifestyle intervention for specific populations (South Asian). Canada: Consider lipid screening ‘earlier’ than age 40 y in men and women.
Modified FRS^230^	Population cohort, USA	0%	2008 NICE Guideline: Lipid Modification, Cardiovascular risk assessment and the modification of blood lipids for the prevention of cardiovascular disease	FRS x 1.4 (South Asian men). No suggestion for South Asian women.
QRISK^231^	Electronic medical database, UK	0%	N/A	
QRISK Lifetime/3rd Joint British Societies’ (JBS3) Risk Calculator^27,232^	Electronic medical database, UK	2.3% 0.3% Bangladeshi; 1.0% Indian; 0.5% Pakistani; 0.5% Other Asian	2014 Joint British Societies Recommendations on the Prevention of Cardiovascular Disease	
QRISK2^233^	Electronic medical database, UK	1.11% 0.16% Bangladeshi (0.26% Women, 0.17% Men); 0.48% Indian (0.47% Women, 0.48% Men); 0.26% Pakistani (0.26% Women, 0.27% Men); 0.21% Other Asian (0.16% Women, 0.19% Men)	2014 NICE Guideline: Lipid Modification, Cardiovascular risk assessment and the modification of blood lipids for the prevention of cardiovascular disease^230^	
QRISK3^234^	Electronic medical database, UK	5.25% 0.9% Bangladeshi (0.8% Women, 1.1% Men); 2.0% Indian (1.9% Women, 2.1% Men); 1.1% Pakistani (1.0% Women, 1.2% Men); 1.25% Other Asian (1.3% Women, 1.2% Men)	N/A	
PREDICT^30^	Electronic medical database, New Zealand	“Indian”: 9% (5% Women, 4% Men)	2018 Cardiovascular Disease Risk Assessment and Management for Primary Care^235^	For “South Asian peoples” (Indian, including Fijian Indian, Sri Lankan, Afghani, Bangladeshi, Nepalese, Pakistani, Tibetan), begin CVD risk assessment for men at age 30 y and in women aged 40 y, 15 y earlier than other population groups in New Zealand.
INTERHEART Modifiable Risk Score^236^	Cases of MI, age (±5 y), and sex-matched control, 52 countries	14%	N/A	
NORRISK2^237^	Population-based survey cohort, Norway	0%	2017 Norwegian Directorate of Health, National guidelines for prevention of cardiovascular disease	Not validated among South Asian immigrants in Norway. NORRISK2 x 1.4 for all South Asians.
NORRISK2-SADia^238^	Updated NORRISK2; Population-based survey cohort, Norway.	12% South Asian (5% Women, 7% Men)	N/A	Developed to include South Asian adults and those with diabetes.
ETHRISK^239^	Recalibrated FRS, using 2 community-based surveys, UK.	46% 11% Bangladeshi; 20% Indian; 15% Pakistani	N/A	Developed for British black and ‘minority ethnic’ groups without established diabetes or cardiovascular disease.
UK Prospective Diabetes Study (UKPDS)^240^	Population Cohort, UK	“Asian-Indian”: 10% (3% Women, 7% Men)	N/A	CVD risk estimate for adults with type 2 diabetes mellitus.

a If applicable.

ACC = American College of Cardiology; AHA = American Heart Association; CVD = cardiovascular disease; EAS = European Atherosclerosis Society; ESC = European Society of Cardiology; FRS = Framingham Risk Score; MI = myocardial infarction; N/A = not applicable; NICE = National for Health and Clinical Excellence; UK = United Kingdom; USA = United States of America; WHO = World Health Organization.

**TABLE 4:** Summary of Advanced Measures of CAC in South Asian Adults

Author	Study	Main Finding(s)
Kanaya et al^109^	MASALA, MESA	South Asian and NHW men have similar CAC burden (mean age 58 ± 9y vs 63 ± 10 y), but higher CAC burden compared to other race/ethnic groups (mean age 62 ± 10y). CAC scores were similar for South Asian women compared to all women of other racial/ethnic groups; however, South Asian women >70 y had a higher prevalence of any CAC than most other racial/ethnic groups.
Al Rifai et al^254^	MASALA, MESA	South Asian adults have a higher number of vessels with calcified plaque compared to other racial/ethnic groups, OR (95% CI): 0.29 (0.17, 0.40) (*P* < 0.05). Compared to NHW, South Asian adults have significantly lower CAC volume [beta coefficient (95% CI), −0.46 (−0.62, −0.29)] but higher overall CAC density [beta coefficient (95% CI)], 0.14 (0.11, 0.18)]. South Asian adults had higher (OR, 95% CI) overall [0.07 (0.03, 0.12)] and RCA [0.09 (0.03, 0.16)] CAC density compared to other racial/ethnic groups.
Roos et al^250^	Observational Cohort	Compared to White adults, South Asian adults patients had a significantly higher CAC score and higher prevalence of significant CAD (41% vs 28%, respectively, *P* = 0.008), involving more coronary vessels and segments. Significant CAD (>50% stenosis) was more frequent in the left anterior descending coronary artery among asymptomatic South Asian adults compared to White adults with T2D.
Al Rifai et al^249^	MASALA	The PCE was associated with both CAC density [beta coefficient (95% CI), 0.24 (0.12, 0.35)] and CAC volume (beta coefficient (95% CI), 0.43 (0.38, 0.48). HDL-C was directly associated with CAC density and waist circumference was inversely associated with CAC density (*P* < 0.05). Body mass index, hypertension status, statin use, diabetes, and HOMA-IR were all directly associated with CAC volume (*P* < 0.05).
Kanaya et al^264^	MASALA, MESA	Age-adjusted CAC incidence was similar in South Asian men compared with White, Black, and NHW men, but significantly higher than Chinese men (11.1% vs 5.7%, *P* = 0.008). There was no difference in CAC incidence or progression between South Asian women and women of other racial/ethnic groups.
Bhatia et al^255^	MASALA	CAC volume and density were highest, and incident CAC was most common in the left anterior descending artery. Highest volume change was in the right coronary artery. Highest change in density was in the left main coronary artery. Smoking is associated with CAC volume progression. Lipoprotein(a) and exercise are associated with CAC density progression.
Patel et al^169^	MASALA, MESA	Compared to other racial/ethnic groups, the presence of an FamHx in South Asian adults is associated with CAC > 300, OR (95% CI): 2.82 (1.6-4.93). The presence of an FamHx provides significant information for the prediction and reclassification of severe CAC in South Asians: *c*-statistic increase from 0.853 to 0.863 (*P* = 0.001), net reclassification improvement 38.9% (95% CI: 14.6%-62.6%].
Wang et al^170^	SABRE	South Asian adults with FamHx had a trend toward increased CAC burden compared with Europeans, OR 95% CI: 1.28, 0.85-1.93 (*P* = 0.243).

CAC = coronary artery calcium; CI = confidence interval; FamHx = family history of CHD; HOMA-IR = homeostatic model assessment for insulin resistance; MASALA = Mediators of Atherosclerosis in South Asians Living in America;NHW = non-Hispanic White; OR = odds ratio; PCE = pooled cohort equations; SABRE = South Hall and Brent Revisited study; T2D = type 2 diabetes.

## HIGHLIGHTS

- South Asians experience a higher burden of CHD compared with adults of other racial/ethnic groups.
- Culturally adapted assessment and management of traditional and nontraditional risk factors is essential.
- Available population ASCVD risk assessment tools may be unreliable, particularly for those at borderline and intermediate risk; CAC testing may help refine risk and personalize care.
- Evaluation of South Asian subpopulations will help characterize the contribution of social determinants, environmental influences, and genetic susceptibility on heterogenous cardiovascular disease burden.

## ABBREVIATIONS AND ACRONYMS

ACC: American College of Cardiology
ADA: American Diabetes Association
AHA: American Heart Association
ASCVD: atherosclerotic cardiovascular disease
BMI: body mass index
BP: blood pressure
CAC: coronary artery calcium
CHD: coronary heart disease
CVD: cardiovascular disease
EHR: electronic health record
GDM: gestational diabetes mellitus
HDL-C: high-density lipoprotein cholesterol
HDP: hypertensive disorders of pregnancy
LDL-C: low-density lipoprotein-cholesterol
MI: myocardial infarction
NHW: non-Hispanic White
PCE: pooled cohort equations
SA: South Asian
T2D: type 2 diabetes
WHO: World Health Organization
