# Supplementary material for: Identification and Management of Atherosclerotic Cardiovascular Disease Risk in South Asian Populations in the U.S
Source: JACC Adv. Author manuscript; Available in PMC 2023 Dec 12. (PMC10715803; doi:10.1016/j.jacadv.2023.100258)
Supplement: Supplemental Material [file NIHMS1934835-supplement-Supplemental_Material.docx]

| Supplemental Table 1: South Asian ancestry and increased cardiovascular risk – a synthesis of evidence | | | | | |
| --- | --- | --- | --- | --- | --- |
| Author | Name of Study/cohort | Study design | Age group | Years of recruitment | Findings |
| South Asian Countries | | | | | |
| Roth et al.^1^ | Global burden of disease Study (2019) | Cross sectional study of mortality, disability, injuries and risk factors in 145 countries. These included high, medium and low-income counties. | NA | 2019 | The proportion of total deaths due to CVD was highest in China (43%) followed by India (27.4%) and Pakistan (22.7%). However, Indian and Pakistan had a higher prevalence of premature death when compared with China. |
| Joseph et al.^2^ | Prospective Urban Rural Epidemiology (PURE) | Global cohort study with 155,722 participants without CVD, including 33,583 from India, Bangladesh or Pakistan. | 35-70 years | 2003-2014 | Bangladesh had the highest incidence of CVD (17.07, 95% CI 5.06 -19.08; per 1000 person years) followed by Pakistan (9.74, 95% CI 7.91 -11.57; 1000 person years) and India (4.35, 95% CI 4.14 -4.56; per 1000 years) |
| Joshi et al^3^ | The Effect of Potentially Modifiable Risk Factors Associated with Myocardial Infarction (INTERHEART) | Global case control study including 1732 patients presenting with the first diagnosis of an AMI, and 2204 controls. Included patients from 5 SA countries. | NA | 1999-2003 | Mean age of first AMI was nearly 10 years lower in South Asian countries (53 years) vs Western Europe and China (63 years) |
| United States | | | | | |
| Kanaya et al.^4^ | Mediators of Atherosclerosis in South Asians Living in America (MASALA) Study | US based cohort study of ~900 SA living in the Chicago and Bay area. | 40-79 | 2010-2018 | SA men had faster progression of coronary artery calcium compared with Black, Latino and Chinese Americans. No significant differences were noted among women. |
| Satish et.al.^5^ | National health interview survey (NHIS) | A cross sectional US based study evaluating the prevalence of self-reported ASCVD and its risk factors among disaggregated Asian subgroups and NHW. | 18 years or older | 2007-2019 | Asian Indian men had a lower age and sex adjusted odds for ASCVD when compared with NHW (OR 0.49, 95% CI 0.44 – 0.55). However, when adjusted for sociodemographic factors and cardiovascular risk factors, no differences were seen between the groups. Underdiagnosis of ASCVD may have led to lower event rates in Asian Indians in the study. |
| Manjunath et.al.^6^ | Palo Alto Medical Foundation | EMR based retrospective cohort study evaluating treatment patterns and incidence of AMI after the diagnosis of CAD in various ethnic subgroups within the US. | 67 (mean) | 2006-2015 | Asian Indians had a higher risk of having an AMI (17.4%) within 1 year of coronary angiography when compared with NHW (11.2%) (OR 1.7, 95% CI 1.21-2.34). |
| Shah et al.^7^ | The National Center for Health Statistics | Mortality trends in the US were studied among disaggregated Asian American subgroups. | NA | 2003-2017 | Asian Indian Men had a slower decline in age standardized mortality rates (ASMR) from IHD and heart failure, compared with NHW. Asian Indian women had an increase in ASMR from heart failure during this period. |
| Enas et al.^8^ | Self-reported survey among Asian Indian physicians compared with the Framingham Offspring study | The prevalence of CHD and its risk factors was compared with the native Caucasian population | 20 years or older | 1990- 1996 | Asian Indian men had an approximately 3 times higher risk for acute myocardial infarction or angina when compared with the Framingham Offspring study. (7.2% versus 2.5%; P < 0.0001). There was no significant difference noted among women. |
| Klatsky et al.^9^ | Northern California health system database using self-reported race | Rates of hospitalization for CHD were compared between different ethnic subgroups and compared White individuals. | 30-40 (mean) | 1978-1985 | Indians and Pakistanis had an RR of 3.7 for hospitalization for CHD when compared with the white population. |
| Canada | | | | | |
| Anand et al.^10^ | Study of Health Assessment and Risk in Ethnic groups (SHARE) study | Cross sectional study of 342 SA, compared with Chinese (317) and European (326) individuals in Canada. | 30-70 | 1996-1998 | SA had a higher prevalence of established CVD (8.6%) vs Chinese (1.9%) and European individuals (4.9%). For any given atherosclerotic burden, SA had more events than the other two groups. |
| Tu et al.^11^ | Cardiovascular Health in Ambulatory Care Research Team (CANHEART)  Immigrant Study | Retrospective population based cohort studying the risk of a major CV event among various immigrant subgroups in Canada. | 30-74 | 1985-2000 | Higher combined incidence of AMI, Stroke, revascularization and cardiovascular death in SA men (8.9%) vs other immigrant groups (2.4- 7.2%), driven mainly by AMI. Similar results were seen in SA (3.6%) vs other immigrant women (1.1-2.9%) |
| Europe | | | | | |
| Fedeli et al.^12^ | Veneto, Italy;  Multiple data sources including chronic disease registry, hospital discharge records, and medication databases. | Prevalence of cardiovascular risk factors and disease was compared between immigrants ( based on country of origin) and locals. | 20-59 | 2015 | SA had the highest SMR for CHD death among all immigrant groups assessed. SA men: SMR 2.3 compared to locals. SA women: SMR 4.53 compared with locals. |
| Satish et al.^13^ | Catalan Health Surveillance System, Spain | Retrospective cohort study using disaggregated data from 71,100 SA and 5.3 million native individuals in Catalonia, Spain. | 18 years or older | 2011-2019 | The highest prevalence of CHD was seen in Bangladeshi men (7.3%), and Pakistani women (3.2%). Indian men had the lowest prevalence of CHD across all SA groups. |
| Patel et al.^14^ | UK Bio Bank study | UK based prospective cohort study of individuals without ASCVD including 8124 individuals of SA ancestry. | 40-69 | 2006-2010 | Over 11 years follow up, SA had twice the risk of having an ASCVD event compared with European individuals (HR of 2.03, 95% CI, 1.86–2.22; P<0.001). Both PCE and QRISK 3 calculators do not fully capture the increased CVD risk in this group. |
| Tan et al.^15^ | The London Life Sciences Prospective Population Study (LOLIPOP) | UK based cohort study including ~24000 SA and European men and women. | 35-75 | 2002-2008 | Higher prevalence of age and sex adjusted CHD (OR 2.55, 95% CI 2.26-2.87, p< 0.001) in SA participants when compared with European individuals. |
| Wild et al.^16^ | Census data from England and Wales | Standardized mortality ratios (SMR) were calculated for deaths from different causes in immigrants with disaggregation at the level of the country. | 20 years or older | 2001-2003 | SMR for circulatory death was highest in Bangladeshi immigrants when compared with all groups. For CHD among SA subgroups- Bangladeshi immigrants had the highest SMR followed by Pakistani) and Indian immigrants. |
| Regidor et al.^17^ | Data from the municipal population register and the cause of death register in Madrid, Spain | Age adjusted mortality from cardiovascular disease was calculated for men from different immigrant groups and compared with men born in Spain. | 20-64 | 2001-2005 | SA men had the highest mortality rates from cardiovascular disease with a mortality rate ratio of 2.8 when compared with the Spanish population. |
| Hippley-Cox et al.^18^ | General practices in England enrolled in the QResearch database | Prospective cohort study designed to develop and validate the QRISK3 score | 25-84 | 1998-2015 | Compared to local Whites, Pakistani women had a 70% higher CVD risk while Bangladeshi and Indian men had a 70% higher CVD risk. |
| George et. al^19^ | Clinical Disease Research using Linked Bespoke studies and Electronic health Records (CALIBER) program | Cohort study evaluating 1,068,318 patients free of CVD. This included predominantly white (90%) but 3.6% SA and 2.9% Black individuals. | 30 years or older | 1997-2010 | Compared to White individuals, SA were about 10 years younger at the initial diagnosis of CVD. SA had a higher risk for CAD (1.73, 95% CI 1.51 -1.98) when compared with White individuals. |
| Tillin et al.^20^ | Southall and Brent Revisited (SABRE) study | London based cohort study of 1517 SA vs European vs African Caribbean men and women without known heart disease. | 40-69 | 1991-1998 | At 13 year follow up, SA had a higher incidence of heart disease (age and sex adjusted HR 1.7, 95% CI 1.52-1.91) compared with Europeans. They were also diagnosed 2.3 years earlier than European participants. |
| McKeigue et al.^21^ | Census data from England and Wales | Data was disaggregated by boroughs where individual SA subgroups resided and standardized mortality ratios calculated. | NA | 1981 | Asian men and women uniformly had a 50% greater CHD mortality than the national average. This was despite significant risk factor differences between Gujarati, Punjabi and Bangladeshi boroughs. |

SA- South Asian; AMI- acute myocardial infarction, HR- hazard ratio; OR – odds ratio; RR- relative risk; SMR - Standardized mortality ratios; NHW – non Hispanic white; CVD – cardiovascular disease; CHD- coronary heart disease; ASCVD – atherosclerotic cardiovascular disease; CAD – coronary artery disease; IHD – ischemic heart disease; PCE – Pooled cohort equations; UK - United Kingdom; US – United States; NHIS – National Health Interview Survey; EMR – Electronic medical record; NA – Not applicable

1. Zhao D. Epidemiological Features of Cardiovascular Disease in Asia. *JACC Asia.* 2021;1(1):1-13.

2. Joseph P, Kutty VR, Mohan V, et al. Cardiovascular disease, mortality, and their associations with modifiable risk factors in a multi-national South Asia cohort: a PURE substudy. *Eur Heart J.* 2022;43(30):2831-2840.

3. Joshi P, Islam S, Pais P, et al. Risk factors for early myocardial infarction in South Asians compared with individuals in other countries. *JAMA.* 2007;297(3):286-294.

4. Kanaya AM, Vittinghoff E, Lin F, et al. Incidence and Progression of Coronary Artery Calcium in South Asians Compared With 4 Race/Ethnic Groups. *J Am Heart Assoc.* 2019;8(2):e011053.

5. Satish P, Sadaf MI, Valero-Elizondo J, et al. Heterogeneity in cardio-metabolic risk factors and atherosclerotic cardiovascular disease among Asian groups in the United States. *Am J Prev Cardiol.* 2021;7:100219.

6. Manjunath L, Chung S, Li J, Shah H, Palaniappan L, Yong CM. Heterogeneity of Treatment and Outcomes Among Asians With Coronary Artery Disease in the United States. *J Am Heart Assoc.* 2020;9(10):e014362.

7. Shah NS, Xi K, Kapphahn KI, et al. Cardiovascular and Cerebrovascular Disease Mortality in Asian American Subgroups. *Circ Cardiovasc Qual Outcomes.* 2022;15(5):e008651.

8. Enas EA, Garg A, Davidson MA, Nair VM, Huet BA, Yusuf S. Coronary heart disease and its risk factors in first-generation immigrant Asian Indians to the United States of America. *Indian Heart J.* 1996;48(4):343-353.

9. Klatsky AL, Tekawa I, Armstrong MA, Sidney S. The risk of hospitalization for ischemic heart disease among Asian Americans in northern California. *Am J Public Health.* 1994;84(10):1672-1675.

10. Anand SS, Yusuf S, Vuksan V, et al. Differences in risk factors, atherosclerosis, and cardiovascular disease between ethnic groups in Canada: the Study of Health Assessment and Risk in Ethnic groups (SHARE). *Lancet.* 2000;356(9226):279-284.

11. Tu JV, Chu A, Rezai MR, et al. The Incidence of Major Cardiovascular Events in Immigrants to Ontario, Canada: The CANHEART Immigrant Study. *Circulation.* 2015;132(16):1549-1559.

12. Fedeli U, Avossa F, Ferroni E, et al. Diverging patterns of cardiovascular diseases across immigrant groups in Northern Italy. *Int J Cardiol.* 2018;254:362-367.

13. Satish P, Vela E, Bilal U, et al. Burden of cardiovascular risk factors and disease in five Asian groups in Catalonia: a disaggregated, population-based analysis of 121 000 first-generation Asian immigrants. *Eur J Prev Cardiol.* 2021.

14. Patel AP, Wang M, Kartoun U, Ng K, Khera AV. Quantifying and Understanding the Higher Risk of Atherosclerotic Cardiovascular Disease Among South Asian Individuals: Results From the UK Biobank Prospective Cohort Study. *Circulation.* 2021;144(6):410-422.

15. Tan ST, Scott W, Panoulas V, et al. Coronary heart disease in Indian Asians. *Glob Cardiol Sci Pract.* 2014;2014(1):13-23.

16. Wild SH, Fischbacher C, Brock A, Griffiths C, Bhopal R. Mortality from all causes and circulatory disease by country of birth in England and Wales 2001-2003. *J Public Health (Oxf).* 2007;29(2):191-198.

17. Regidor E, Astasio P, Calle ME, Martinez D, Ortega P, Dominguez V. The association between birthplace in different regions of the world and cardiovascular mortality among residents of Spain. *Eur J Epidemiol.* 2009;24(9):503-512.

18. Hippisley-Cox J, Coupland C, Brindle P. Development and validation of QRISK3 risk prediction algorithms to estimate future risk of cardiovascular disease: prospective cohort study. *BMJ.* 2017;357:j2099.

19. George J, Mathur R, Shah AD, et al. Ethnicity and the first diagnosis of a wide range of cardiovascular diseases: Associations in a linked electronic health record cohort of 1 million patients. *PLoS One.* 2017;12(6):e0178945.

20. Tillin T, Hughes AD, Mayet J, et al. The relationship between metabolic risk factors and incident cardiovascular disease in Europeans, South Asians, and African Caribbeans: SABRE (Southall and Brent Revisited) -- a prospective population-based study. *J Am Coll Cardiol.* 2013;61(17):1777-1786.

21. McKeigue PM, Marmot MG. Mortality from coronary heart disease in Asian communities in London. *BMJ.* 1988;297(6653):903.

| **Supplemental** **Table 2a:** List of published studies in South Asian adults. | | |
| --- | --- | --- |
| **Name of study** | **Study description** | **Findings** |
| South Asian Heart Risk Assessment^1^ (SAHARA) (NCT03309579) | RCT studying the effect of a culturally tailored digital health intervention targeting diet and physical exercise in SA without heart disease. | A culturally tailored digital intervention did not show a significant change in MI risk score (a combination of cardiac risk factors) after 1 year, when compared with placebo. |
| Change of Fructose to Fat in South Asians^2^ (NCT01562782) | An interventional non randomized study evaluating the differences in denovo lipogenesis with fructose consumption in SA vs Caucasians. | Hepatic denovo lipogenesis after a fructose load was higher in young SA compared with Caucasians despite normal indices of insulin sensitivity. |
| The GlasVEGAS Study (Glasgow Visceral & Ectopic Fat With Weight Gain in South AsianS^3^  (NCT02399423) | An interventional non randomized study evaluating ethnic differences in fat metabolism after weight gain and weight loss. Investigators compared SA and European men at the start of the study, after 7% weight gain, and again after 7-15% weight loss (from peak weight). | Initial report showed a decrease in whole body insulin sensitivity by 38% with ~6.4% weight gain in SA but not in white Europeans. SA had more total fat, higher subcutaneous, deep subcutaneous and liver fat, but not visceral fat. There was a correlation between insulin resistance and deep subcutaneous fat in SA, this depot was larger and metabolically similar to visceral fat. |
| South Asian Birth Cohort (START)^4^ | The START cohort followed pregnant women and their children in rural/urban India, and in Canada to study environmental and genetic determinants of adiposity in this group. | SA women were younger and had a higher prevalence of gestational diabetes (21% vs 13%) compared with white women. SA newborns had a lower birth weight, greater skinfold thickness and waist circumference compared with white newborns. |

1. Anand SS, Samaan Z, Middleton C, et al. A Digital Health Intervention to Lower Cardiovascular Risk: A Randomized Clinical Trial. *JAMA Cardiol.* 2016;1(5):601-606.

2. Hudgins LC, Hugo JL, Enayat S, Parker TS, Artis AS, Levine DM. Young, healthy South Asians have enhanced lipogenic sensitivity to dietary sugar. *Clin Endocrinol (Oxf).* 2017;86(3):361-366.

3. McLaren J. Adiposity and diabetes risk mechanisms in South Asians. *University of Glasgow* 2019.

4. Anand SS, Gupta MK, Schulze KM, et al. What accounts for ethnic differences in newborn skinfold thickness comparing South Asians and White Caucasians? Findings from the START and FAMILY Birth Cohorts. *Int J Obes (Lond).* 2016;40(2):239-244.

| **Supplemental** **Table 2b:** List of ongoing studies in South Asian adults, by activity status. | | |
| --- | --- | --- |
| **Name of study** | **Study Design and Purpose** | **Expected Completion** |
| **Studies that are actively recruiting** | | |
| A Culturally-tailored Personalized Nutrition Intervention in South Asian Women at Risk of Gestational Diabetes (DESI-GDM) (NCT03607799) | A randomized study to assess the impact of a culturally tailored, personalized nutrition intervention on glycemic response to an oral glucose load in high-risk pregnancies of SA women. | 2023 |
| Novel Model for South Asian Treatment in Diabetes (NaMaSTe-Diabetes) Trial in Primary Care (NCT02136654) | RCT evaluating a culturally tailored lifestyle and medication adherence intervention in SA patients with poorly controlled Diabetes. | 2021 |
| Effects of Glucose Lowering Agents in South Asian Women With Impaired Glucose Tolerance or Impaired Fasting Glucose (DIASA3) (NCT04662866) | RCT evaluating the efficacy of Metformin, Empaglazoin, Piaglitazone and Linagliptin on hepatic insulin sensitivity in SA women with impaired glucose tolerance or impaired fasting glucose. | 2023 |
| Effects of Substituting Sitting With Standing and Light Intensity Activity in Free-living Conditions on Glycaemia in Overweight and Obese South Asian Adults (NCT04645875) | RCT of a SitLess regimen of sitting for minimum 5 hours, >2 hours light physical activity and > 3 hours of standing vs a Sit regimen (< 2 hours standing) will improve glycaemia in overweight and obese SA adults. | 2021 |
| South Asians and coronary plaque registry (NCT05367297) | A registry of individuals who self-report as South Asian ethnicity with coronary plaque assessment compared with non- SA controls. | 2027 |
| Better Together Study  (NCT05275231) | This study seeks to test that a culturally-tailored 16-week lifestyle intervention in immigrant men from South Asia and West Africa. | 2023 |
| Pakistan Study of Premature Coronary Atherosclerosis in Young Adults (PAKSEHAT)  (NCT05156736) | A cohort study of 2000 asymptomatic Pakistani men and women without clinical CVD. These participants will receive a CTA at baseline and will be followed up for 5 years to assess plaque progression and for clinical events. | 2027 |
| Comparison of Optimal Hypertension Regimens (AIMHY-INFORM)  (NCT02847338) | RCT comparing the effect of 4 antihypertensive on automated office blood pressure measurement in SA: Amlodipine, Lisinopril, Amiloride and Chlorthalidone. | 2022 |
| Secular Trends in the Prevalence of Cardio metabolic Risk Factors Among Teenage School Children in Urban South India  (NCT04015726) | A cross sectional study conducted in 22 schools Chennai, India to assess the prevalence of cardiovascular risk factors in children aged 12-18 years. | 2021 |
| South Asians Active Together (SAATH) Study (NCT04400253) | RCT testing the effectiveness and implementation of a multi-component physical activity intervention directed at the environment, family, interpersonal and individual levels to promote physical activity among immigrant SA mothers and daughters. | 2024 |
| **Studies that are active but not recruiting** | | |
| Integrated Community-Clinical Linkage Model to Promote Weight Loss Among South Asians With Pre-Diabetes  (NCT03188094) | A study assessing the effectiveness and implementation of an integrated community and primary care based intervention to support weight loss for SA patients at risk for type II diabetes. | 2023 |
| Prevention of Type 2 Diabetes Amongst South Asians With Central Obesity and Prediabetes (iHealth-T2D) (NCT02949739) | An RCT assessing if intensive lifestyle modification treatment reduces the risk of type II diabetes in SA with central obesity and pre diabetes. | 2022 |
| South Asian Arrhythmogenic Cardiomyopathy Registry  (NCT04895540) | A registry of patients with arrhythmogenic cardiomyopathy patients and their affected relatives across two sites in India. Includes phenotypic and genetic analysis. | 2022 |
| Diabetes Management Intervention For South Asians  (NCT03333044) | An RCT to assess a multi-level integrated intervention for SA with uncontrolled diabetes. The primary outcome of is a reduction in hemoglobin A1c levels with the intervention vs standard of care. | 2024 |
| Generalizability of REDUCE-IT Results to People of South Asian Descent With Atherosclerotic Cardiovascular Disease in Canada (REDUCE-IT Canada SA) (NCT05271591) | Observational study assessing the generalizability of the REDUCE IT trial to the SA population in Canada. The study will evaluate the proportion of SA individuals who have indication for Icosapent ethyl, alignment with the RECDUCE IT cohort as well as access to coverage. | 2022 |
| Chronic Cardiovascular Risk Outpatient Management in South Asians Using Digital Health Technology HealthPals (NCT03167996) | The study will assess the use of telemedicine and mobile health to track health behavior, facilitate communication between providers and SA patients, and provide virtual health coaching. The outcomes include a change in exercise activity, medication compliance, compliance to dietary recommendations, blood pressure trends, and changes in weight. | 2018 |
| South Asian Healthy Lifestyle Initiative (SAHELI) (NCT03336255) | A pilot study designed to study the feasibility and initial efficacy of a culturally targeted community based lifestyle intervention on physical activity and diet behaviors among medically underserved SA | 2023 |
| CTA - Computed tomography angiography; CVD - Cardiovascular disease; MI –myocardial infarction; SA- South Asians; RCT – Randomized controlled trials. | | |
